# OATP1B1/1B3 deficiency exacerbates hyperbilirubinemia in erythropoietic protoporphyria

**DOI:** 10.1016/j.dmd.2025.100105

**Published:** 2025-05-27

**Authors:** Ruizhi Gu, Fu-Ying Qin, Luxuan Wang, Jiaojiao Zhang, Jacob Emerson, Qing Ma, Jie Lu, Karl E. Anderson, Junmei Wang, Xiaochao Ma

**Affiliations:** 1 Center for Pharmacogenetics, Department of Pharmaceutical Sciences, School of Pharmacy, University of Pittsburgh, Pittsburgh, Pennsylvania; 2 Computational Chemical Genomics Screening Center, Department of Pharmaceutical Sciences, School of Pharmacy, University of Pittsburgh, Pittsburgh, Pennsylvania; 3 Department of Pharmacy Practice, School of Pharmacy and Pharmaceutical Sciences, University at Buffalo, Buffalo, New York; 4 Porphyria Laboratory & Center, Division of Gastroenterology and Hepatology, Department of Internal Medicine, University of Texas Medical Branch, Galveston, Texas

**Keywords:** OATPs, Protoporphyrin IX, Bilirubin, Liver diseases

## Abstract

Erythropoietic protoporphyria (EPP) is caused by loss-of-function mutations in ferrochelatase (FECH), leading to the accumulation of its substrate, protoporphyrin IX (PPIX). PPIX is primarily produced in the bone marrow and transported to the liver for excretion. Because PPIX is hydrophobic, its elevated levels can cause bile duct blockage, cholestatic liver injury, and even liver failure. However, the specific transporter responsible for PPIX uptake into hepatocytes remains unclear. The OATP1B1/1B3 transporters, which are expressed in hepatocytes, facilitate the uptake of coproporphyrin III, a structural analog of PPIX. Additionally, OATP1B1/1B3 mediates the uptake of bilirubin, a biomarker of liver injury, from plasma into the liver for excretion. Therefore, we aimed to determine the role of OATP1B1/1B3 in regulating PPIX and bilirubin homeostasis under EPP conditions. A mouse strain carrying a Fech mutation was used as an EPP model. Building on this, we generated a new EPP mouse model with Oatp1a/1b deficiency. Using these EPP mouse models, along with OATP1B1/1B3-overexpressing cells, our study revealed that PPIX is not a substrate of OATP1B1/1B3. Notably, our work found that genetic deficiency or pharmacologic suppression of Oatp1a/1b exacerbates hyperbilirubinemia in EPP mice without worsening liver injury. Mechanistically, Oatp1a/1b deficiency impairs bilirubin uptake from plasma, while Fech deficiency leads to PPIX-mediated bile duct blockage and reduced bilirubin excretion, synergistically exacerbating hyperbilirubinemia. In summary, our work demonstrated that deficiency or suppression of Oatp1a/1b exacerbates hyperbilirubinemia in EPP mouse models, suggesting that assessment of OATP1B1/1B3 function is crucial in EPP patients with EPP with hyperbilirubinemia.

**Significance Statement:**

This work revealed that serum bilirubin levels are not paralleled with liver damage in the erythropoietic protoporphyria mouse models with Oatp1a/1b deficiency. Our findings suggest that assessment of OATP1B1/1B3 function is crucial in patients with erythropoietic protoporphyria with hyperbilirubinemia.

## 1 Introduction

Erythropoietic protoporphyria (EPP) is an inherited disorder caused by loss-of-function mutations in ferrochelatase (FECH), the last enzyme in the heme biosynthesis pathway that converts protoporphyrin IX (PPIX) to produce heme.^1^, ^2^, ^3^ These mutations lead to PPIX accumulation in red blood cells, plasma, and the liver.^4^, ^5^, ^6^, ^7^ The elimination of PPIX from the body relies on the hepatobiliary system.^6^^,^^7^ Because PPIX is highly hydrophobic, excessive PPIX will precipitate in bile ducts, resulting in bile duct blockage and cholestatic liver injury in patients with EPP.^6^^,^^8^ EPP-associated cholestatic liver injury can progress to liver failure, which is fatal without liver transplant.^9^^,^^10^

Due to the shortage of liver donors, the model for end-stage liver disease (MELD) score is used to prioritize patients in need of liver transplants.^11^ Total serum bilirubin is one of the variables to calculate the MELD score and a biomarker of liver injury.^11^^,^^12^ Bilirubin is a breakdown metabolite of heme, which also relies on the hepatobiliary system for elimination.^13^^,^^14^ Although total serum bilirubin is important for the calculation of the MELD score, there are confounding factors that can disturb bilirubin homeostasis, potentially leading to misjudgments in liver transplant prioritization.

Organic anion transporting polypeptides (OATPs) facilitate bilirubin uptake from plasma into hepatocytes for elimination.^15^, ^16^, ^17^ In humans, OATP1B1 and OATP1B3, homologous to mouse Oatp1b2, are key transporters involved in hepatic bilirubin uptake.^18^^,^^19^ Loss-of-function mutations in OATP1B1 and OATP1B3 are linked to Rotor syndrome, a genetic disorder characterized by bilirubin accumulation in the blood.^18^ Similarly, elevated bilirubin levels have been observed in Oatp1a/1b-deficient mice.^17^ Furthermore, many clinically used drugs can inhibit OATP1B1/1B3, leading to increased bilirubin levels in the blood.^20^ These findings raise concerns about the impact of OATP1B1/1B3 deficiency on bilirubin homeostasis and liver function in the context of EPP.

Beyond their role in bilirubin uptake, the involvement of OATP1B1/1B3 in PPIX uptake from plasma into hepatocytes remains unclear. PPIX is primarily synthesized in the bone marrow and transported in the bloodstream by red blood cells.^4^^,^^5^ Once released into plasma, PPIX is taken up by hepatocytes and eliminated via the hepatobiliary system.^7^ However, excessive PPIX accumulation in the bile ducts can lead to blockage and cholestatic liver injury.^6^^,^^8^ Therefore, hepatic uptake transporters for PPIX may play a crucial role in modulating EPP-associated liver injury. Human OATP1B1/1B3, which are exclusively expressed in hepatocytes, are responsible for the uptake of coproporphyrin III (CopIII), a structural analog of PPIX,^21^^,^^22^ suggesting that OATP1B1/1B3 may also contribute to PPIX uptake in hepatocytes.

This study aimed to address 3 key questions regarding OATP1B1/1B3 and EPP: (1) How does OATP1B1/1B3 deficiency affect bilirubin homeostasis in the context of EPP? (2) Is PPIX a substrate of OATP1B1/1B3? and (3) Does OATP1B1/1B3 deficiency influence liver injury in EPP, and if so, by what mechanism? A mouse strain with a loss-of-function mutation of Fech (Fech-mut) was used as an EPP model.^23^^,^^24^ Additionally, we developed an Oatp1a/1b-deficient mouse model on the Fech-mut background (Fech-mut/Oatp1a/1b-knockout [KO]). Using these models, along with pharmacological inhibitors of OATP1B1/1B3, and OATP1B1/1B3-overexpressing cell lines, we systematically explored the role of OATP1B1/1B3 in bilirubin and PPIX homeostasis, as well as its impact on liver injury in EPP.

## 2 Methods and methods

### 2.1 Chemicals and reagents

Methanol, acetonitrile, PPIX, bilirubin, and the bilirubin analysis kit were purchased from Sigma-Aldrich. Rifampicin (RIF) was obtained from TCI Chemical, while CopIII was sourced from Frontier Scientific Inc. Kits for serum alanine aminotransferase (ALT), aspartate aminotransferase (AST), and alkaline phosphatase (ALP) analyses were purchased from Pointe Scientific Inc.

### 2.2 EPP mouse models and treatment

Fech-mut mice were developed by a loss-of-function mutation of Fech.^23^^,^^24^ Oatp1a/1b-KO mice were originally generated in Dr Schinkel’s laboratory.^17^ Fech-mut/Oatp1a/1b-KO mice were generated by crossbreeding Fech-mut mice with Oatp1a/1b-KO mice. These mouse models were verified by genotyping. The expression levels of Oatp1a1, 1a4, and 1b2 in the liver were further confirmed by quantitative real-time polymerase chain reaction (qPCR) analysis. Mice between 8 and 12 weeks old were used for analyzing serum metabolome, bilirubin homeostasis, PPIX distribution, and liver functions. Aged mice (20–23 weeks old) were also included for the evaluation of liver injury. The current work focused on male mice because no sex differences of bilirubin or PPIX metabolism and disposition were observed in our preliminary studies. To determine the impact of Oatp1a/1b inhibition on bilirubin homeostasis, Fech-mut mice and Fech-mut/Oatp1a/1b-KO mice were treated orally with RIF (120 mg/kg), and blood samples were collected before and at 2 hours after RIF treatment. All animal studies were approved by the Institutional Animal Care and Use Committee of the University of Pittsburgh.

### 2.3 qPCR analysis

The methods for total mRNA extraction from the liver and reverse transcription to cDNA were the same as previously reported.^25^ cDNA was then used for SYBR Green–based qPCR analysis (Applied Biosystems). Cyclophilin was used as a housekeeping gene. Primers for qPCR analysis are listed in Supplemental Table 1.

### 2.4 Western blot

Liver protein was extracted using radio-immunoprecipitation buffer, and protein concentrations were determined using the Pierce BCA Protein Assay Kit (Thermo Fisher Scientific). Equal amounts of protein (20 *μ*g per sample) were separated on an 8% SDS-polyacrylamide gel and subsequently transferred to polyvinylidene difluoride membranes. The membranes were then incubated with a primary antibody against uridine diphosphate glucuronosyltransferase 1A1 (Ugt1a1) (Abcam, ab62600).

### 2.5 Serum metabolomics

Metabolomics was conducted for the sera from Fech-mut and Fech-mut/Oatp1a/1b-KO mice. In brief, 20 *μ*L of serum was mixed with 80 *μ*L methanol/acetonitrile (1:1, v/v) and then centrifuged at 20,000 × *g* for 10 minutes. Two microliters of supernatant was injected into an ultra-performance liquid chromatography coupled with quadrupole time-of-flight mass spectrometer (Waters Corp) for metabolite analysis. The mass data were first acquired by MassLynx 4.1 (Waters Corporation) and then exported into SIMCA-P (Umetrics) for orthogonal partial least squares discriminant analysis. A score plot and a loading S-plot were generated and used for screening of the metabolites that contributed to the discrimination between Fech-mut and Fech-mut/Oatp1a/1b-KO groups. Collision energy ramping from 15 to 45 V was used for the structural elucidation of metabolites.

### 2.6 PPIX measurement

PPIX levels were analyzed in serum, liver, and bile. Briefly, 20 *μ*L of serum was added into 80 *μ*L of methanol/acetonitrile (1:1, v/v). The mixture was vortexed and the supernatant was collected after centrifuge at 16,300 × *g* for 10 minutes. Liver samples were homogenized in water (50 mg liver in 250 *μ*L water). One hundred microliters of liver homogenate was added in 300 *μ*L of methanol/acetonitrile (1:1, v/v), followed by vortex and centrifuge at 16,300 × *g* for 10 minutes. Bile samples were collected from the gallbladder, and 2 *μ*L of bile was mixed with 300 *μ*L of acetonitrile/isopropanol/water (1:1:1, v/v/v). The mixture was vortexed for 1 minute and then centrifuged at 16,300 × *g* for 10 minutes. Two microliter of supernatant was injected into the ultra-performance liquid chromatography coupled with quadrupole time-of-flight mass spectrometer system for PPIX analysis.

### 2.7 Evaluation of liver injury

Biochemical analyses were conducted to measure serum ALT, AST, ALP, and total serum bilirubin. For histological analysis, liver tissues were fixed in 4% formaldehyde phosphate solution overnight, followed by dehydration and embedding in paraffin. Four-micrometer sections were cut and stained with H&E or Picro-Sirius Red.

### 2.8 Cell-based uptake assays

OATP1B1-, OATP1B3-, and OATP2B1-overexpressing cells (HEK293 cell line), as well as the corresponding control cell lines, were kindly provided by Dr Jörg König (Friedrich-Alexander-Universität). All cells were cultured in the minimum essential media supplemented with 10% fetal calf serum and 5% penicillin/streptomycin. The procedure of uptake assays of CopIII and PPIX was modified from a previous report.^21^ Briefly, 1 × 10^6^ cells were seeded on a poly-D-lysine coated 6-well plate. After seeding for 48 hours, cells were washed and equilibrated in Hanks’ balanced salt solution/10 mM HEPES (pH 7.4). Cells were then treated with 10 *μ*M of CopIII or PPIX dissolved in the buffer indicated above. After 10 or 30 minutes of incubation at 37 °C, the culture media with CopIII or PPIX were removed, and cells were gently washed 3 times. Next, cells were lysed by 1% Triton X-100/1 mM HEPES and 100 *μ*L of supernatants were transferred into a 96-well black plate for fluorescence analysis. CopIII was measured at *λ*_ex_ 395 nm/*λ*_em_ 620 nm, and PPIX was measured at *λ*_ex_ 405 nm/*λ*_em_ 630 nm.^26^^,^^27^

### 2.9 Statistics

One-way ANOVA and 2-tailed Student’s *t* test were conducted using GraphPad Prism 9.5 (GraphPad). A *P* value <.05 was considered statistically significant.

## 3 Results

### 3.1 Genetic deficiency of Oatp1a/1b causes jaundice in EPP mice

**Fig. 1:**
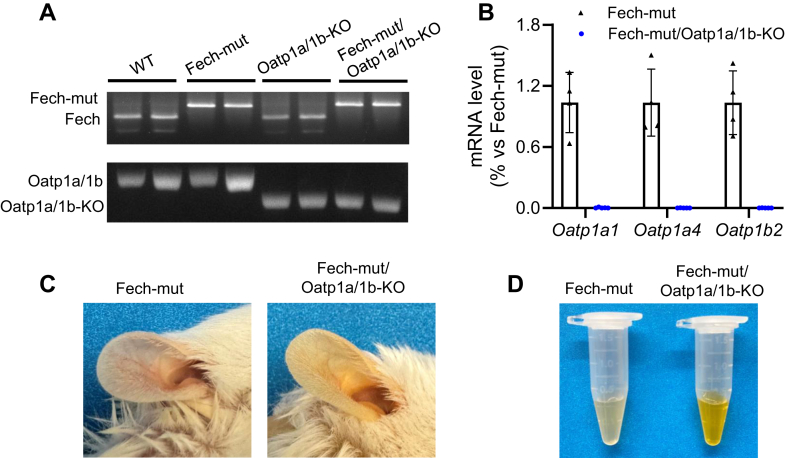
Characterization of Fech-mut/Oatp1a/1b-KO mice. (A) Genotyping results of WT, Fech-mut, Oatp1a/1b-KO, and Fech-mut/Oatp1a/1b-KO mice. (B) Hepatic expression of Oatp1a/1b genes, quantified by qPCR. Expression levels in Fech-mut mice are set to 1, with data presented as mean ± SD (*n* = 4–5). (C) Gross appearance of mouse ears. (D) Serum samples from Fech-mut and Fech-mut/Oatp1a/1b-KO mice. The ear skin and serum of Fech-mut/Oatp1a/1b-KO mice (right) appear more yellow compared to Fech-mut mice (left).

Oatp1a/1b-KO mice were backcrossed with Fech-mut mice for 3 generations to generate Fech-mut/Oatp1a/1b-KO mice. Genotyping confirmed that Fech-mut/Oatp1a/1b-KO mice are deficient in both Fech and Oatp1a/1b (Fig. 1A). Additional qPCR analysis verified the deficiency of Oatp1a1, Oatp1a4, and Oatp1b2 genes in the liver of Fech-mut/Oatp1a/1b-KO mice (Fig. 1B). Notably, Fech-mut/Oatp1a/1b-KO mice exhibited more pronounced jaundice in both their skin and serum compared to Fech-mut mice (Fig. 1, C and D).

### 3.2 Metabolomic analysis reveals exacerbated hyperbilirubinemia in EPP mice with Oatp1a/1b deficiency

**Fig. 2:**
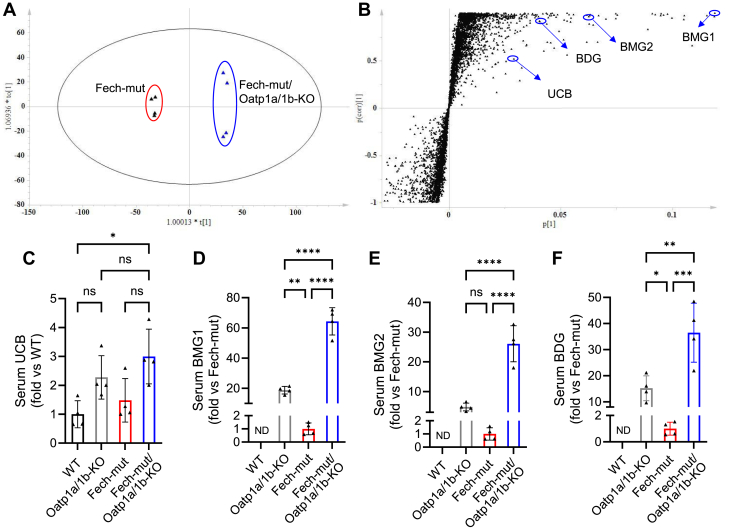
Metabolomic analysis reveals hyperbilirubinemia in Fech-mut/Oatp1a/1b-KO mice. (A, B) Metabolomic analysis of serum samples from Fech-mut and Fech-mut/Oatp1a/1b-KO mice. (A) Score plot illustrating the separation between Fech-mut and Fech-mut/Oatp1a/1b-KO groups. (B) Loading S-plot highlighting metabolites contributing to group separation. The *x*-axis represents the relative abundance of metabolites, while the *y*-axis indicates the correlation of each metabolite to the model. UCB, BMG1, BMG2, and BDG were among the top-ranking metabolites in Fech-mut/Oatp1a/1b-KO mouse sera. (C–F) Relative quantification of UCB, BMG1, BMG2, and BDG in serum. Data are presented as mean ± SD (*n* = 4). ∗*P* < .05; ∗∗*P* < .01; ∗∗∗*P* < .001; ∗∗∗∗*P* < .0001. ND, not detected; ns, not significant.

The serum metabolomes of Fech-mut and Fech-mut/Oatp1a/1b-KO mice were clearly separated in a score plot (Fig. 2A). Unconjugated bilirubin (UCB) and conjugated bilirubin (CBs) were among the top-ranking ions contributing to the separation between Fech-mut and Fech-mut/Oatp1a/1b-KO mice (Fig. 2B, Supplemental Fig. 1). Compared to the wild-type (WT) group, serum UCB levels were increased by approximately 2.2-, 1.5-, and 3.0-fold in Oatp1a/1b-KO, Fech-mut, and Fech-mut/Oatp1a/1b-KO mice, respectively (Fig. 2C). Notably, serum levels of CBs, including bilirubin monoglucuronide (BMG) 1, BMG2, and bilirubin diglucuronide (BDG) were significantly higher in Fech-mut/Oatp1a/1b-KO mice compared to the other strains (Fig. 2, D–F). Specifically, serum BMG1 levels were 3.4- and 64.3-fold higher in Fech-mut/Oatp1a/1b-KO mice compared to Oatp1a/1b-KO and Fech-mut mice, respectively (Fig. 2D). Serum BMG2 increased by 5.4- and 26.1-fold in Fech-mut/Oatp1a/1b-KO mice compared to Oatp1a/1b-KO and Fech-mut mice, respectively (Fig. 2E). Serum BDG was elevated by 2.3- and 36.5-fold in Fech-mut/Oatp1a/1b-KO mice compared to Oatp1a/1b-KO and Fech-mut mice, respectively (Fig. 2F). These results indicate that Oatp1a/1b deficiency exacerbates hyperbilirubinemia in EPP mice, with the primary contribution coming from CBs.

### 3.3 Pharmacological inhibition of Oatp1a/1b elevates bilirubin levels in EPP mice

**Fig. 3:**
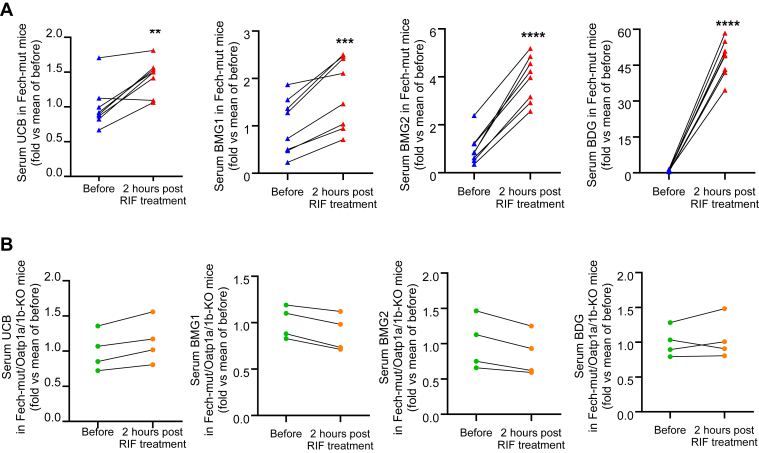
Pharmacological inhibition of Oatp1a/1b increases serum bilirubin levels in Fech-mut mice. Blood samples were collected before and 2 hours after RIF treatment. (A) Relative quantification of serum UCB, BMG1, BMG2, and BDG in Fech-mut mice (*n* = 8) before and after RIF treatment. (B) Relative quantification of serum UCB, BMG1, BMG2, and BDG in Fech-mut/Oatp1a/1b-KO mice (*n* = 4) before and after RIF treatment. For each group, baseline serum levels of UCB, BMG1, BMG2, and BDG before RIF treatment are set to 1. Data were analyzed using a paired *t* test. ∗∗*P* < .01; ∗∗∗*P* < .001; ∗∗*P* < .0001.

To assess the impact of pharmacological suppression of Oatp1a/1b on bilirubin homeostasis in EPP, RIF, a known Oatp1a/1b inhibitor,^28^ was administered to Fech-mut mice. As anticipated, RIF treatment led to a significant increase in serum bilirubin levels in Fech-mut mice, particularly for BMG1, BMG2, and BDG (Fig. 3A). No substantial change in serum bilirubin was observed in Fech-mut/Oatp1a/1b-KO mice following RIF treatment (Fig. 3B), suggesting that the RIF-induced elevation of serum bilirubin is Oatp1a/1b-dependent.

### 3.4 PPIX is not a substrate of OATP1B1/1B3

**Fig. 4:**
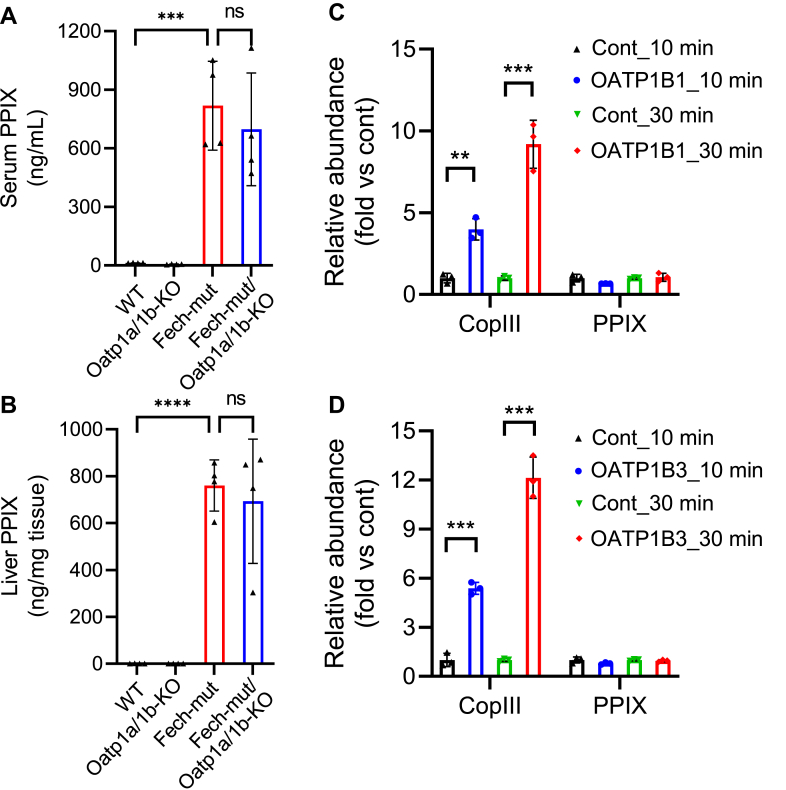
PPIX is not a substrate of OATP1A/1B. (A, B) PPIX levels in the serum (A) and liver (B) of WT, Oatp1a/1b*-*KO, Fech-mut, and Fech-mut/Oatp1a/1b*-*KO mice (*n* = 4 per group). PPIX was analyzed by ultra-performance liquid chromatography coupled with quadrupole time-of-flight mass spectrometer. (C, D) The uptake of PPIX by cells overexpressed with OATP1B1 (C) or OATP1B3 (D). The cells (*n* = 3 per group) were incubated with PPIX (10 *μ*M) for 10 or 30 minutes. CopIII (10 *μ*M), a known substrate of OATP1B1 and 1B3, was used as a positive control. CopIII and PPIX were determined by fluorescence analysis. The data in control groups (C, D) are set as 1. All data are expressed as mean ± SD. ∗∗*P* < .01; ∗∗∗*P* < .001; ∗∗∗∗*P* < .0001. ns, not significant.

As expected, a significant accumulation of PPIX was observed in the serum and liver of Fech-mut mice (Fig. 4, A and B). However, no significant difference in serum or liver PPIX levels was found between Fech-mut and Fech-mut/Oatp1a/1b-KO mice (Fig. 4, A and B), suggesting that Oatp1a/1b deficiency does not affect PPIX distribution. The role of OATPs in PPIX distribution was further investigated using OATP1B1- and OATP1B3-overexpressing cells. Intracellular levels of CopIII, a positive control for OATP1B1/1B3 assays,^21^^,^^22^ were significantly increased in cells overexpressing OATP1B1 or OATP1B3 when incubated with CopIII. However, no significant change in PPIX levels was observed under the same incubation conditions (Fig. 4, C and D). In addition to OATP1B1 and OATP1B3, the role of OATP2B1 in PPIX uptake was also evaluated using CopIII as a positive control.^21^ As expected, CopIII uptake was increased in OATP2B1-overexpressing cells, whereas no change was observed for PPIX (Supplemental Fig. 2). These findings indicate that PPIX is not a substrate of OATP1B1, OATP1B3, or OATP2B1.

### 3.5 Deficiency of Oatp1a/1b does not potentiate liver injury in EPP mice

**Fig. 5:**
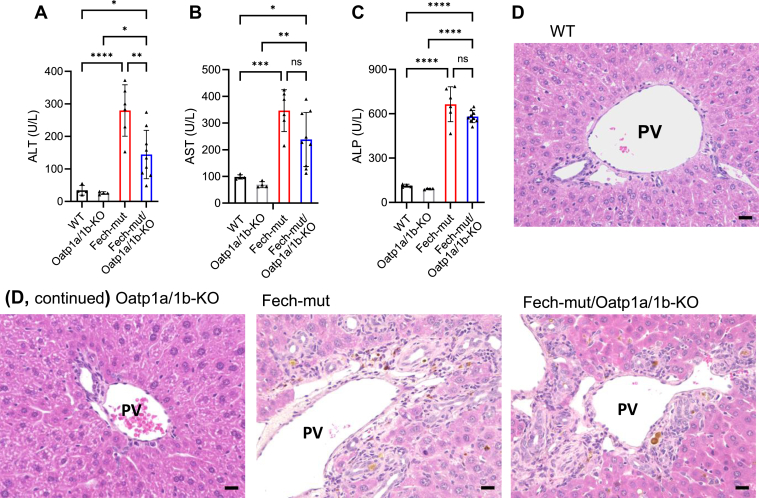
Oatp1a/1b deficiency does not potentiate liver injury in EPP mice. Blood and liver samples were collected from WT, Fech-mut, Oatp1a/1b-KO, and Fech-mut/Oatp1a/1b-KO mice. (A–C) Serum activities of ALT, AST, and ALP. The data are expressed as mean ± SD (*n* = 4–9). ∗*P* < .05; ∗∗*P* < .01; ∗∗∗*P* < .001; ∗∗∗∗*P* < .0001. (D) Representative liver sections with H&E staining. Scale bars, 20 *μ*m. ns, not significant; PV, portal vein.

Liver injury was observed in both Fech-mut and Fech-mut/Oatp1a/1b-KO mice, as demonstrated by biochemical and histological analyses (Fig. 5, Supplemental Fig. 3). Interestingly, serum activities of ALT and AST, biomarkers of hepatocellular injury, were lower in Fech-mut/Oatp1a/1b-KO mice compared to Fech-mut mice, while serum levels of ALP, a biomarker of cholestatic injury, showed no significant difference (Fig. 5, A–C). Consistent with the ALP data, PPIX-induced bile plugs and bile duct blockage were observed in both Fech-mut and Fech-mut/Oatp1a/1b-KO mice, but not in WT or Oatp1a/1b-KO mice (Fig. 5D). Additionally, Fech-mut and Fech-mut/Oatp1a/1b-KO mice exhibited similar levels of liver fibrosis (Supplemental Fig. 3). Moreover, liver injury markers were not elevated in aged Fech-mut/Oatp1a/1b-KO mice compared to age-matched Fech-mut mice (Supplemental Fig. 4). These findings suggest that Oatp1a/1b deficiency does not exacerbate EPP-associated cholestatic liver injury in mice.

### 3.6 Mechanisms of hyperbilirubinemia in EPP mice with Oatp1a/1b deficiency

**Fig. 6:**
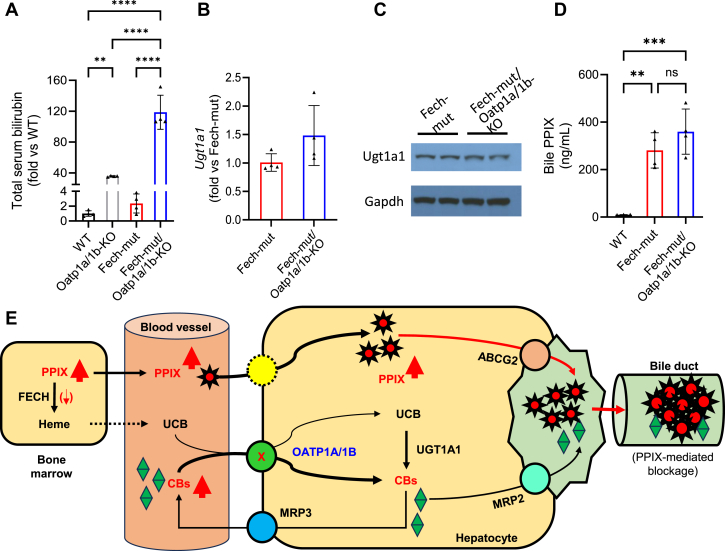
Deficiency of Oatp1a/1b exacerbates hyperbilirubinemia in EPP mouse models. (A) Total bilirubin levels in the serum of WT, Oatp1a/1b-KO, Fech-mut, and Fech-mut/Oatp1a/1b-KO mice. (B) qPCR analysis of Ugt1a1 mRNA in the liver of Fech-mut and Fech-mut/Oatp1a/1b-KO mice. (C) Western blot of Ugt1a1 protein in the liver of Fech-mut and Fech-mut/Oatp1a/1b-KO mice. (D) PPIX levels in the bile of WT, Fech-mut, and Fech-mut/Oatp1a/1b-KO mice. Data are expressed as mean ± SD (*n* = 3 or 4). ∗∗*P* < .01; ∗∗∗*P* < .001; ∗∗∗∗*P* < .0001. (E) A proposed mechanism of hyperbilirubinemia in EPP mice with Oatp1a/1b deficiency: deficiency of Oatp1a/1b impairs the uptake of both UCB and CBs from the plasma. At the same time, PPIX-mediated bile duct blockage through ABCG2 reduces the hepatobiliary excretion of CBs, resulting in a synergistic effect that leads to hyperbilirubinemia. ns, not significant.

Compared to WT mice, total serum bilirubin levels increased approximately 36-, 2-, and 119-fold in Oatp1a/1b-KO, Fech-mut, and Fech-mut/Oatp1a/1b-KO mice, respectively (Fig. 6A). To investigate the underlying mechanisms contributing to the exacerbated hyperbilirubinemia in Fech-mut/Oatp1a/1b-KO mice, we examined key enzymes and transporters involved in bilirubin homeostasis. The hepatic expression of Ugt1a1, the enzyme responsible for converting UCB to CBs,^29^ showed no significant difference between Fech-mut and Fech-mut/Oatp1a/1b-KO mice (Fig. 6, B and C). Similarly, the expression levels of multidrug resistance–associated protein (Mrp) 2, the apical efflux transporter responsible for excreting CBs into bile,^30^ and Mrp3, the sinusoidal efflux transporter that pumps CBs into the bloodstream for reuptake into hepatocytes via OATPs,^31^ were unchanged between Fech-mut and Fech-mut/Oatp1a/1b-KO mice (Supplemental Fig. 5). These findings suggest that the exacerbated hyperbilirubinemia in Fech-mut/Oatp1a/1b-KO mice is not due to altered Ugt1a1, Mrp2, or Mrp3 expression. In addition, we observed a marked increase in PPIX concentration in the bile of Fech-mut and Fech-mut/Oatp1a/1b-KO mice (Fig. 6D), along with evidence of bile duct blockage in the livers of these EPP mouse models (Fig. 5D). These results support the notion that hyperbilirubinemia in Fech-mut/Oatp1a/1b-KO mice arises from a synergistic effect of Oatp1a/1b deficiency and PPIX-mediated bile duct blockage, because Oatp1a/1b deficiency impairs CB uptake from plasma, while PPIX-induced bile duct obstruction slows CB excretion from the liver, collectively leading to increased CB redistribution into the bloodstream (Fig. 6E).

## 4 Discussion

This study demonstrated that genetic deficiency or pharmacologic suppression of Oatp1a/1b exacerbates hyperbilirubinemia in EPP mice. An EPP mouse model with Oatp1a/1b deficiency was developed, which displayed severe jaundice and hyperbilirubinemia. Pharmacological inhibition of Oatp1a/1b also significantly increased serum bilirubin levels in EPP mice. Mechanistically, deficiency or suppression of Oatp1a/1b impairs bilirubin uptake from plasma, and PPIX-mediated bile duct blockage reduces bilirubin excretion from the liver, which synergistically result in hyperbilirubinemia in EPP mice.

The liver plays a central role in maintaining bilirubin homeostasis.^13^ Hepatocytes take up UCB from the bloodstream, conjugate it into water-soluble forms, and excrete these conjugates into the bile ducts.^32^ Obstruction of the bile ducts impairs this excretory process, leading to the accumulation of bilirubin and its conjugates in the blood, making bilirubin a biomarker of cholestatic liver injury. Additionally, hepatocellular injury can also result in hyperbilirubinemia due to impaired function of enzymes and transporters involved in bilirubin metabolism and clearance, such as UGT1A1 and MRP2.^14^^,^^29^ Owing to its strong association with both cholestatic and hepatocellular liver damage, bilirubin is a critical biomarker for liver injury and is used to assess liver transplant priority in patients with liver failure.^11^^,^^33^

Despite hyperbilirubinemia in Fech-mut/Oatp1a/1b-KO mice, Oatp1a/1b deficiency did not potentiate liver damage in EPP mice, suggesting that serum bilirubin levels are not paralleled with liver damage in the EPP condition with Oatp1a/1b deficiency. Loss-of-function mutations of OATP1B1/1B3 have been discovered in humans, and many clinically used drugs have been identified as OATP1B1/1B3 inhibitors,^18^^,^^28^^,^^34^ which could result in hyperbilirubinemia in patients with EPP if encountered. To accurately assess liver damage in patients with EPP with hyperbilirubinemia, we recommend a thorough evaluation of OATP1B1/1B3 function by analyzing genetic polymorphisms and reviewing their history of exposure to OATP1B1/1B3 inhibitors.

Our work also revealed that PPIX is not a substrate of OATP1B1/1B3. The severity of liver injury associated with EPP varies among individuals, and additional factors are thought to contribute to this variability.^8^^,^^35^ Uptake transporters of PPIX may potentiate EPP-associated liver injury by increasing its hepatic distribution, suggesting that regulators of these transporters could contribute to inter-individual differences in susceptibility. Despite the structural similarities between PPIX and CopIII, a known substrate of OATP1B1, OATP1B3, and OATP2B1,^21^ our study demonstrated that PPIX is not transported by these OATPs. These findings indicate that alternative transport mechanisms are responsible for PPIX uptake into hepatocytes. In addition to membrane transporters, plasma proteins such as hemopexin, lipoproteins, and IgG have been shown to bind PPIX,^36^, ^37^, ^38^ potentially facilitating its delivery to the liver. Further research is needed to identify the specific transport mechanisms mediating PPIX uptake from plasma into hepatocytes.

In summary, this study demonstrated that deficiency or suppression of OATP1B1/1B3 exacerbates hyperbilirubinemia in EPP through a synergistic mechanism that impairs bilirubin uptake from the bloodstream and reduces its excretion from the liver. Additionally, our findings indicate that serum bilirubin levels do not necessarily correlate with liver damage in EPP cases with OATP1B1/1B3 deficiency or suppression, suggesting that assessment of OATP1B1/1B3 function is crucial in patients with EPP with hyperbilirubinemia, particularly those being considered for liver transplant. Our findings may also have broader implications for other cholestatic liver diseases.

## Conflict of interest

The authors have no conflicts to report.
