# Supplementary material for: OATP1B1/1B3 deficiency exacerbates hyperbilirubinemia in erythropoietic protoporphyria
Source: Drug Metab Dispos. 2025 May 27;53(7):100105. doi: 10.1016/j.dmd.2025.100105 (PMC12405900; doi:10.1016/j.dmd.2025.100105)
Supplement: Supplementary Table 1 and Supplementary Figures 1-5 [file mmc1.docx]

**Supplemental Material**

**OATP1A/1B deficiency exacerbates hyperbilirubinemia in erythropoietic protoporphyria**

Ruizhi Gu,^1^ Fu-Ying Qin,^1^ Luxuan Wang,^2^ Jiaojiao Zhang,^1^ Jacob Emerson,^1^ Qing Ma,^3^ Jie Lu,^1^ Karl E. Anderson,^4^ Junmei Wang,^2^ Xiaochao Ma^1, *^

**Supplemental Table 1.** Primers for qPCR analysis.

**Supplemental Figure 1.** MS/MS spectra and fragmentations of UCB, BMG1, BMG2, and BDG.

**Supplemental Figure 2.** The uptake of PPIX by cells overexpressed with OATP2B1.

**Supplemental Figure 3.** Sirius Red staining of liver sections from WT, Oatp1a/1b-KO, Fech-mut, and Fech-mut/Oatp1a/1b-KO mice.

**Supplemental Figure 4.** Oatp1a/1b deficiency does not potentiate liver injury in aged EPP mice.

**Supplemental Figure 5.** qPCR analysis of *Abcc2* (Mrp2), *Abcc3* (Mrp3), and *Abcg2* (Bcrp) mRNA in the liver of Fech-mut and Fech-mut/Oatp1a/1b-KO mice.

**Supplemental Table 1.** Primers for qPCR analysis.

| **Gene** | **Forward 5’-3’** | **Reverse 5’-3’** |
| --- | --- | --- |
| ***Oatp1a1*** | TAGCTTGCCTCCAGTATGCCTT | ACAGGCCAAATGCTATGTATGC |
| ***Oatp1a4*** | CAAGCTTTCTCCCTGCACTCTT | TCCTTCGCAGTGAGCTTCATT |
| ***Oatp1b2*** | AGCAATGATCGGACCAATGCT | AACCCAACGAGCATCCTGA |
| ***Abcc2*** | TACCAGCGAGTTATCGAAGCGTG | TGCTTCTGACCGCCACTGAGAT |
| ***Abcc3*** | ACTTCCTCCGAAACTACGCACC | GCTGGCTCATTGTCTGTCAGGT |
| ***Ugt1a1*** | TGTGTGTGTTCGGTCCCTCT | GCTATGACCACAACTTCGTGC |
| ***Cyclophilin*** | GGAGATGGCACAGGAGGAA | GCCCGTAGTGCTTCAGCTT |
| ***Abcg2*** | TGGACTCAAGCACAGCGAAT | ATCCGCAGGGTTGTTGTAGG |

**Supplemental Figure 1**


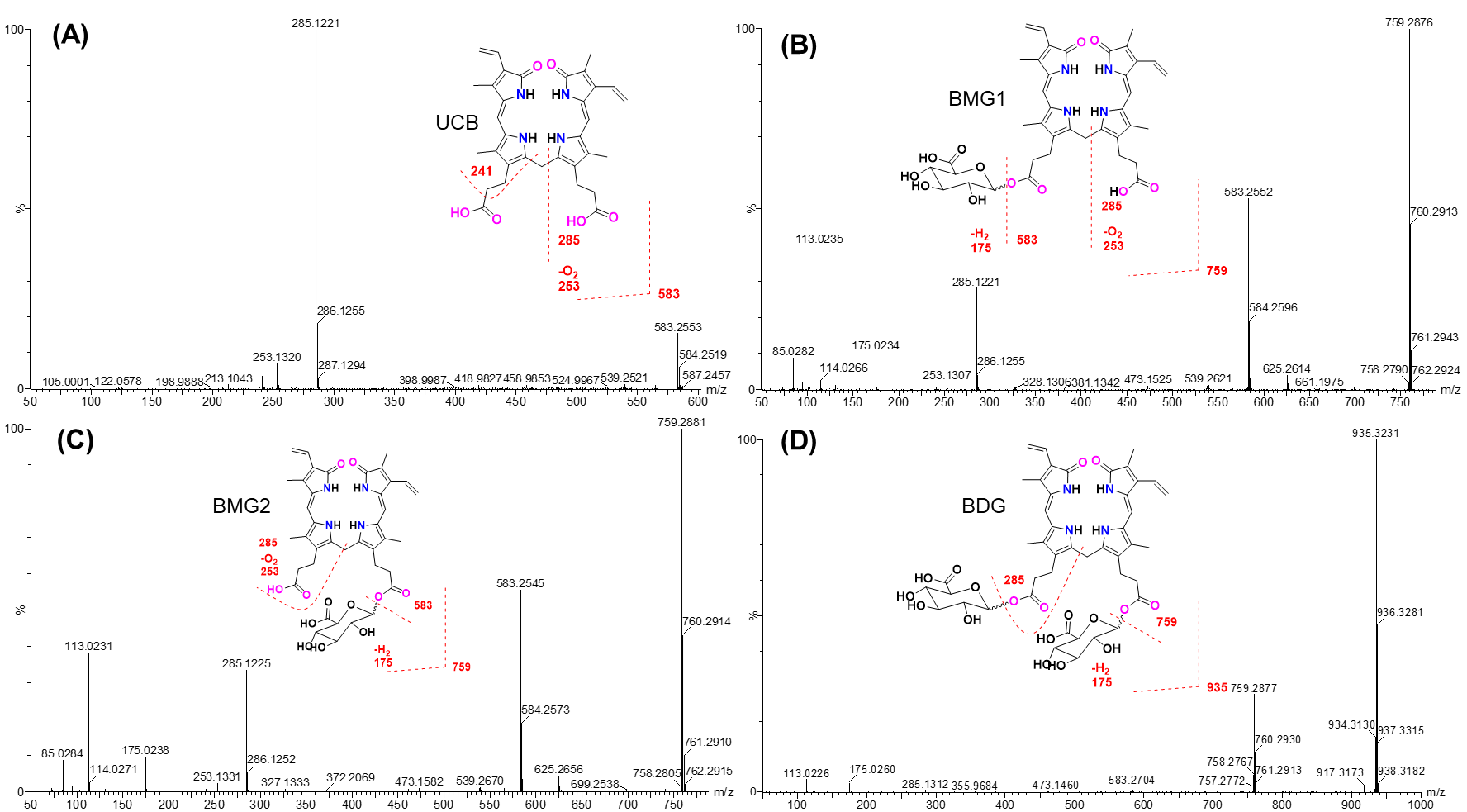


**Supplemental Figure 1.** MS/MS spectra and fragmentations of UCB (A), BMG1 (B), BMG2 (C), and BDG (D). UCB and its metabolites were analyzed by UPLC-QTOFMS.

**Supplemental Figure 2**

**Supplemental Figure 2.** The uptake of PPIX by cells overexpressed with OATP2B1. The cells were incubated with CopIII or PPIX (10 µM) for 30 min. CopIII and PPIX were determined by fluorescence analysis. The data in control groups are set as 1. All data are expressed as mean ± SD (n = 3). **P < 0.01.

**Supplemental Figure 3**


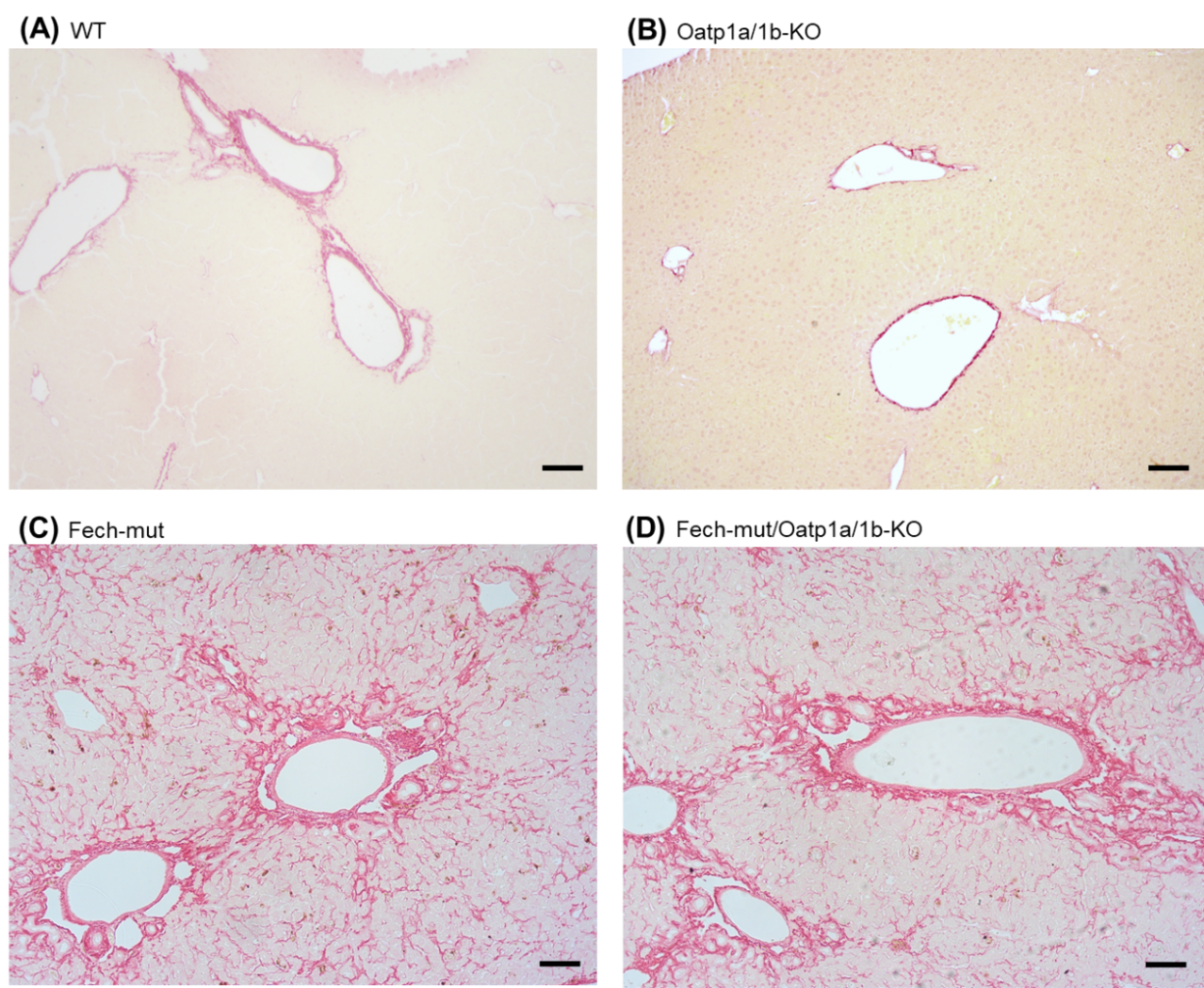


**Supplemental Figure 3.** Sirius Red staining of liver sections from WT (A), Oatp1a/1b*-*KO (B), Fech-mut (C), and Fech-mut/Oatp1a/1b*-*KO (D) mice. Scale bars, 50 μm.

**Supplemental Figure 4**

**
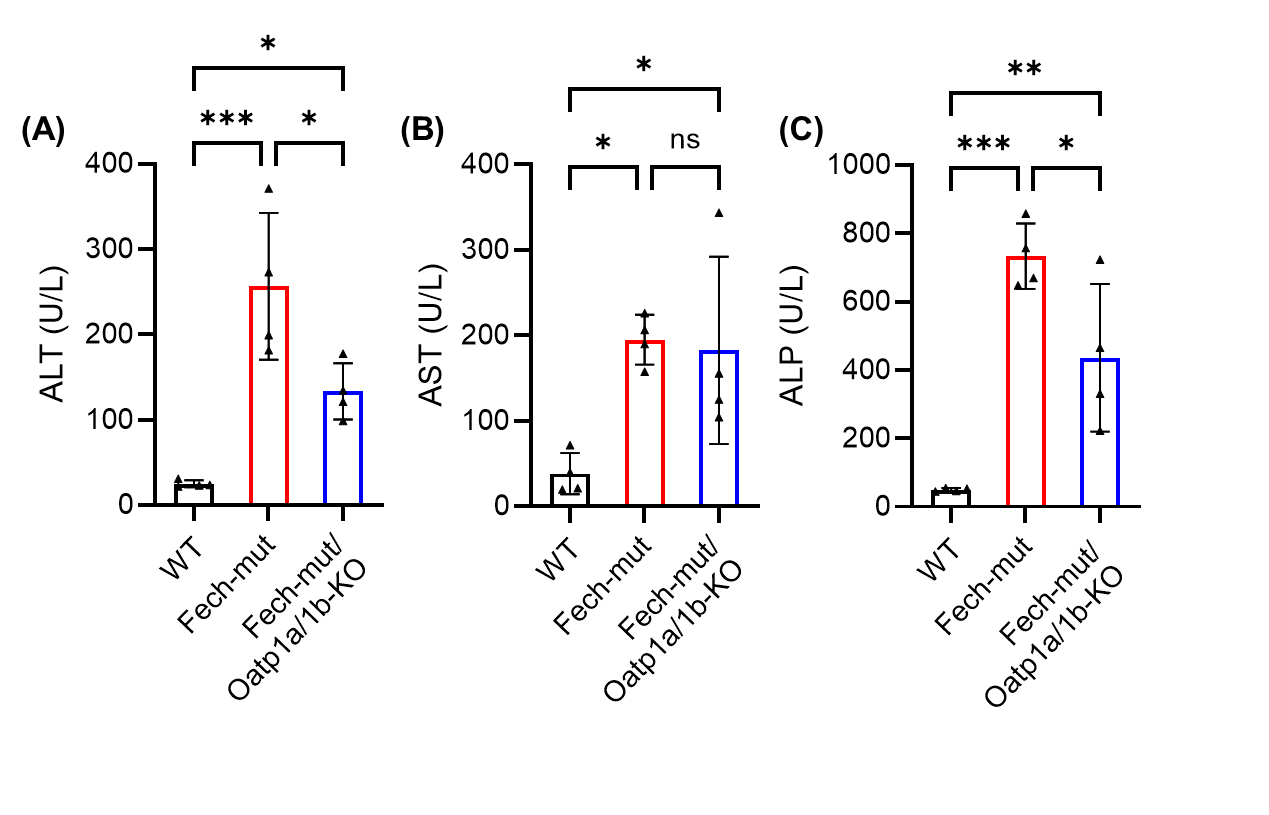
**

**Supplemental Figure 4.** Oatp1a/1b deficiency does not potentiate liver injury in aged EPP mice. Activities of ALT (A), AST (B), and ALP (C) were measured in the serum of WT, Fech-mut, and Fech-mut/Oatp1a/1b-KO mice (20-23 weeks old). The data are expressed as mean ± SD (n = 4). *P < 0.05, **P < 0.01, ***P < 0.001. ns, not significant.

**Supplemental Figure 5**

**
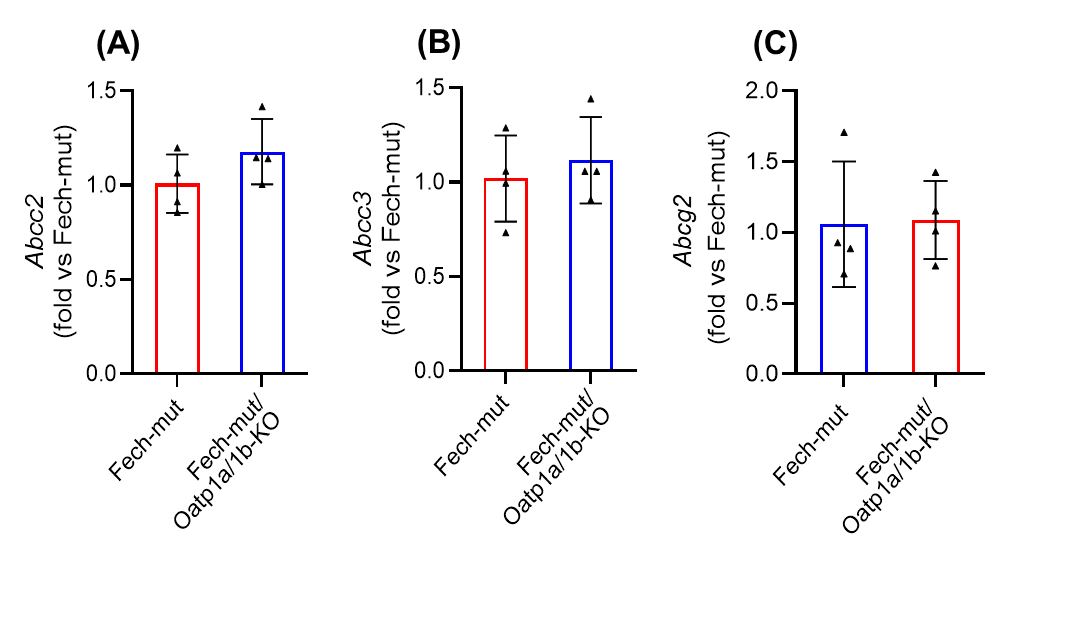
**

**Supplemental Figure 5.** qPCR analysis of *Abcc2* (Mrp2), *Abcc3* (Mrp3), and *Abcg2* (Bcrp) mRNA in the liver of Fech-mut and Fech-mut/Oatp1a/1b-KO mice. Data are normalized to the Fech-mut group (set as 1). All data are presented as mean ± SD (n = 4 per group).
